# Supplementary material for: The yeast mitochondrial porin represses Snf1/AMP kinase signaling to attenuate viral replication
Source: Genetics. 2026 Apr 24;233(3):iyag106. doi: 10.1093/genetics/iyag106 (PMC7619096; doi:10.1093/genetics/iyag106)
Supplement: iyag106_Supplementary_Data [file iyag106_supplementary_data.zip › Supplemental_Figure_Legends_GENETICS-2026-309123.docx]

**Figure S1. Por1 does not regulate L-BC levels in stationary phase.** RT-qPCR quantification of L-B RNA normalized to endogenous *ACT1* RNA in the indicated strains cultured for 7 days. Mean RNA level and standard deviation are shown. n = 3. * *p* < 0.05. The *p* value was calculated using unpaired student’s t-test.

**Figure S2. Por1 represses Snf1 activity specifically in stationary phase.** **(a, b)** Western blotting of phosphorylated Snf1, Snf1-FLAG, Pgk1, and/or Por1 protein levels in the indicated strains. Samples were collected from cultures grown for the indicated time points in YPAD media. Molecular weight markers are indicated on the right. **(c)** Quantification of phosphorylated Snf1 to total Snf1 in indicated strains normalized to Pgk1 protein level. Samples were collected at indicated time points. Relative fold change to wild type and standard deviation are shown. n = 3. * *p* < 0.05, ** *p* < 0.01. The *p* value was calculated using unpaired student’s t-test.

**Figure S3. TCA cycle is not required for L-A replication in stationary phase.** Western blotting of L-A Gag and Pgk1 protein levels in the indicated strains. Samples were collected from 7-day cultures grown in SC media. Molecular weight markers are indicated on the right.

**Figure S4. *por1Δ* causes L-A dependent sensitivity to AZC in post-stationary phase cells.** Spot test growth assays of independently isolated strains of the indicated genotypes are shown. Strains were cultured for 7 days then tested for recovery from stationary phase under the proteotoxic proline analog, azetidine-2-carboxylic acid (AZC). Strains were spotted on SC plates with or without 0.1 mg/mL of AZC and grown at 30°C.
