# Supplementary material for: The yeast mitochondrial porin represses Snf1/AMP kinase signaling to attenuate viral replication
Source: Genetics. 2026 Apr 24;233(3):iyag106. doi: 10.1093/genetics/iyag106 (PMC7619096; doi:10.1093/genetics/iyag106)
Supplement: iyag106_Supplementary_Data [file iyag106_supplementary_data.zip › Table_S1_GENETICS-2026-309123.docx]

| Enzyme | Metabolic Cycle | Requires for L-A Gag Accumulation in *por1Δ* | Subcellular Localization |
| --- | --- | --- | --- |
| Icl1 | Glyoxylate | Yes | Cytosol |
| Mls1 | Glyoxylate | Yes | Cytosol/Peroxisome |
| Dal7 | Glyoxylate | No | Peroxisome |
| Mdh2 | Glyoxylate | Yes | Cytosol/Peroxisome |
| Mdh3 | Glyoxylate | No | Peroxisome |
| Cit2 | Glyoxylate | Yes | Cytosol/Peroxisome |
| Aco2 | Glyoxylate/TCA | No | Mitochondria |
| Fum1 | TCA | Yes | Cytosol/Mitochondria |
| Mdh1 | TCA | No | Mitochondria |
| Mae1 | TCA | No | Mitochondria |
| Cit1 | TCA | No | Mitochondria |
| Idh2 | TCA | No | Mitochondria |
| Lsc1 | TCA | No | Mitochondria |
| Lsc2 | TCA | No | Mitochondria |
| Sdh1 | TCA | Yes | Mitochondria |
| Sdh9 | TCA | No | Mitochondria |

**Table S1. Summary of the effect glyoxylate/TCA cycle has on L-A Gag accumulation in *por1Δ*.** Abundance of L-A Gag was approximated with western blotting. Only genes that completely suppressed L-A Gag level back to that of wild type when deleted were considered as required for L-A Gag accumulation in *por1Δ*. The metabolic cycle and subcellular localization based on GO term annotations of each enzyme is indicated.
