# Supplementary material for: The yeast mitochondrial porin represses Snf1/AMP kinase signaling to attenuate viral replication
Source: Genetics. 2026 Apr 24;233(3):iyag106. doi: 10.1093/genetics/iyag106 (PMC7619096; doi:10.1093/genetics/iyag106)
Supplement: iyag106_Supplementary_Data [file iyag106_supplementary_data.zip › Table_S2_GENETICS-2026-309123.docx]

**Table S2: Strain and plasmid table**

| Identification | Relevant Genotype |
| --- | --- |
| scy408 | BY4741 *MAT*a L-A^+^ |
| scy410 | BY4741 *MAT*a L-A^+^ *por1Δ::NatMX6* |
| scy464 | BY4741 *MAT*a L-A^0^ *por1Δ::HygMX6* |
| scy466 | BY4741 *MAT*α L-A^0^ *por1Δ::HygMX6* |
| scy499 | BY4741 *MAT*a L-A^0^ |
| scy562 | BY4741 *MAT*a L-A^+^ |
| scy563 | BY4741 *MAT*a L-A^+^ *rim15Δ::KanMX6* |
| scy568 | BY4741 *MAT*a L-A^+^ *snf1Δ::HygMX6* |
| scy569 | BY4741 *MAT*a L-A^+^ *rim15Δ::KanMX6 por1Δ::NatMX6* |
| scy650 | BY4741 *MAT*a L-A^+^ *snf1Δ::HygMX6 por1Δ::NatMX6* |
| scy744 | BY4741 *MAT*a L-A^+^ *por1Δ::NatMX6* |
| scy745 | BY4741 *MAT*a L-A^+^ |
| scy802 | BY4741 *MAT*a L-A^+^ |
| scy803 | BY4741 *MAT*a L-A^+^ *snf4Δ::KanMX6* |
| scy804 | BY4741 *MAT*a L-A^+^ *por1Δ::NatMX6* |
| scy805 | BY4741 *MAT*a L-A^+^ *snf4Δ::KanMX6 por1Δ::NatMX6* |
| scy806 | BY4741 *MAT*a L-A^+^ *sip1Δ::KanMX6* |
| scy807 | BY4741 *MAT*a L-A^+^ *sip1Δ::KanMX6 por1Δ::NatMX6* |
| scy808 | BY4741 *MAT*a L-A^+^ *sip2Δ::KanMX6* |
| scy809 | BY4741 *MAT*a L-A^+^ *sip2Δ::KanMX6 por1Δ::NatMX6* |
| scy810 | BY4741 *MAT*a L-A^+^ *gal83Δ::KanMX6* |
| scy811 | BY4741 *MAT*a L-A^+^ *gal83Δ::KanMX6 por1Δ::NatMX6* |
| scy899 | BY4741 *MAT*a L-A^+^ *adr1Δ::KanMX6 por1Δ::NatMX6* |
| scy900 | BY4741 *MAT*a L-A^+^ |
| scy902 | BY4741 *MAT*a L-A^+^ *por1Δ::NatMX6* |
| scy903 | BY4741 *MAT*a L-A^+^ *adr1Δ::KanMX6* |
| scy905 | BY4741 *MAT*a L-A^+^ *cat8Δ::KanMX6 por1Δ::NatMX6* |
| scy907 | BY4741 *MAT*a L-A^+^ *cat8Δ::KanMX6* |
| scy909 | BY4741 *MAT*a L-A^+^ *sip4Δ::KanMX6* |
| scy910 | BY4741 *MAT*α L-A^+^ *sip4Δ::KanMX6 por1Δ::NatMX6* |
| scy1070 | BY4741 *MAT*a L-A^+^ *icl1Δ::KanMX6* |
| scy1072 | BY4741 *MAT*a L-A^+^ *icl1Δ::KanMX6 por1Δ::NatMX6* |
| scy1074 | BY4741 *MAT*a L-A^+^ *fbp1Δ::KanMX6 por1Δ::NatMX6* |
| scy1076 | BY4741 *MAT*a L-A^+^ *fbp1Δ::KanMX6* |
| scy1078 | BY4741 *MAT*a L-A^+^ *por1Δ::NatMX6* |
| scy1079 | BY4741 *MAT*a L-A^+^ |
| scy1082 | BY4741 *MAT*a L-A^+^ *pck1Δ::KanMX6 por1Δ::NatMX6* |
| scy1083 | BY4741 *MAT*a L-A^+^ *pck1Δ::KanMX6* |
| scy1120 | BY4741 *MAT*a L-A^+^ *por1Δ::NatMX6* |
| scy1122 | BY4741 *MAT*a L-A^+^ *mls1Δ::KanMX6 por1Δ::NatMX6* |
| scy1124 | BY4741 *MAT*a L-A^+^ *dal7Δ::KanMX6 por1Δ::NatMX6* |
| scy1126 | BY4741 *MAT*a L-A^+^ *mdh2Δ::KanMX6 por1Δ::NatMX6* |
| scy1128 | BY4741 *MAT*a L-A^+^ *mdh3Δ::KanMX6 por1Δ::NatMX6* |
| scy1130 | BY4741 *MAT*a L-A^+^ *cit2Δ::KanMX6 por1Δ::NatMX6* |
| scy1165 | BY4741 *MAT*a L-A^+^ *SNF1-3xFLAG::KanMX6 por1Δ::NatMX6* |
| scy1166 | BY4741 *MAT*a L-A^+^ *SNF1-3xFLAG::KanMX6* |
| scy1222 | BY4741 *MAT*a L-A^+^ *fum1Δ::KanMX6 por1Δ::NatMX6* |
| scy1559 | BY4741 *MAT*α L-A^+^ *idh2Δ::HygMX6 por1Δ::NatMX6* |
| scy1685 | BY4741 *MAT*a L-A^+^ *sdh1Δ::KanMX6 por1Δ::NatMX6* |
| scy1691 | BY4741 *MAT*a L-A^+^ *sdh9Δ::KanMX6 por1Δ::NatMX6* |
| scy1694 | BY4741 *MAT*α L-A^+^ *aat1Δ::KanMX6 por1Δ::NatMX6* |
| scy1698 | BY4741 *MAT*a L-A^+^ *aat2Δ::KanMX6* |
| scy1700 | BY4741 *MAT*a L-A^+^ *aat2Δ::KanMX6 por1Δ::NatMX6* |
| scy1702 | BY4741 *MAT*a L-A^+^ *agx1Δ::KanMX6 por1Δ::NatMX6* |
| scy1741 | BY4741 *MAT*a L-A^+^ |
| scy1942 | BY4741 *MAT*a L-A^+^ *HIS3 LEU2 MET15 URA3* |
| scy1949 | BY4741 *MAT*a L-A^+^ *HIS3 LEU2 MET15 URA3 por1Δ::NatMX6* |

*All strains are congenic to BY4741: *his3Δ1 leu2Δ0 met15Δ0 ura3Δ0*.
