# Supplementary figures and images for: The yeast mitochondrial porin represses Snf1/AMP kinase signaling to attenuate viral replication
Source: Genetics. 2026 Apr 24;233(3):iyag106. doi: 10.1093/genetics/iyag106 (PMC7619096; doi:10.1093/genetics/iyag106)

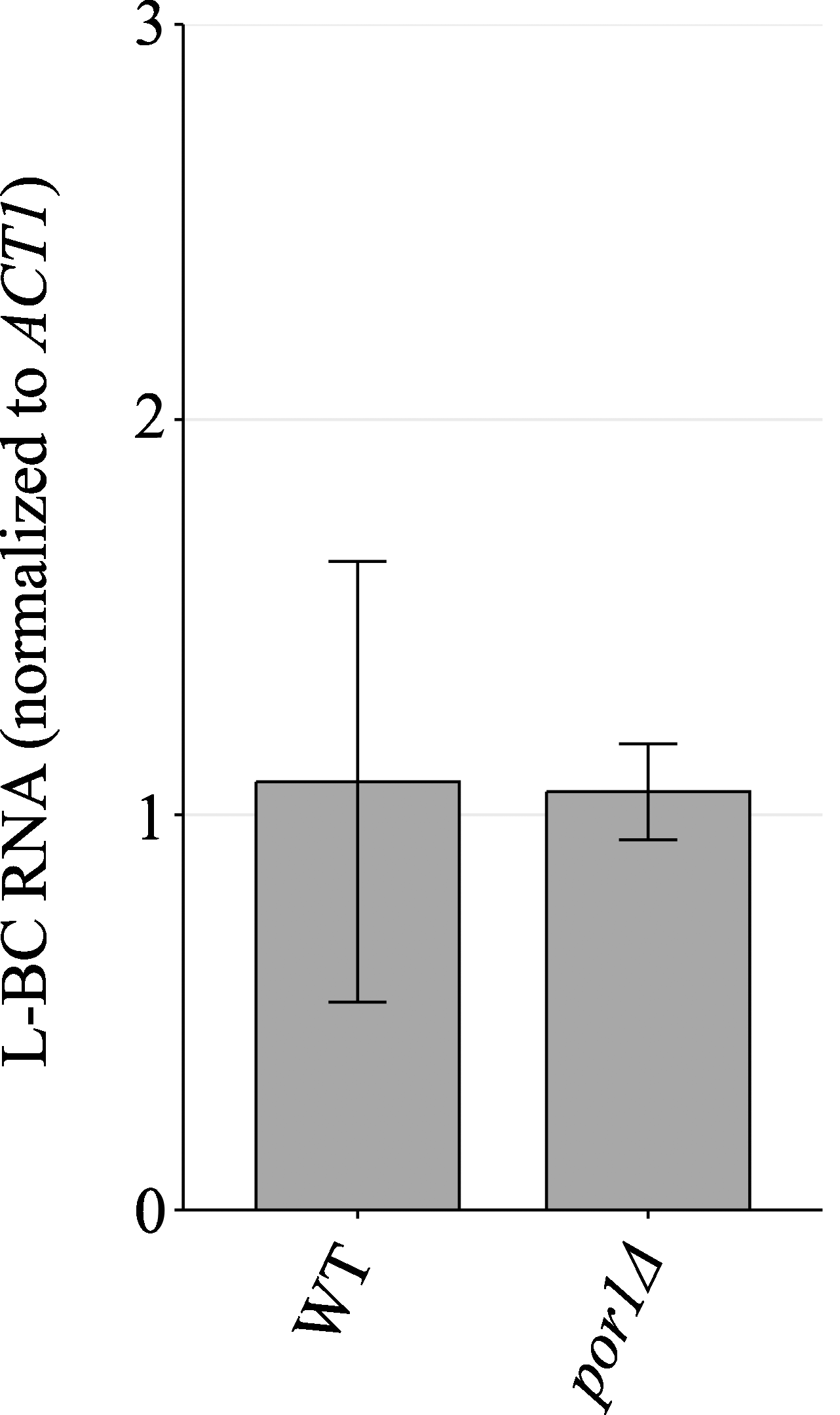

Supplement: iyag106_Supplementary_Data [file iyag106_supplementary_data.zip › Figure_S1_GENETICS-2026-309123.tif]

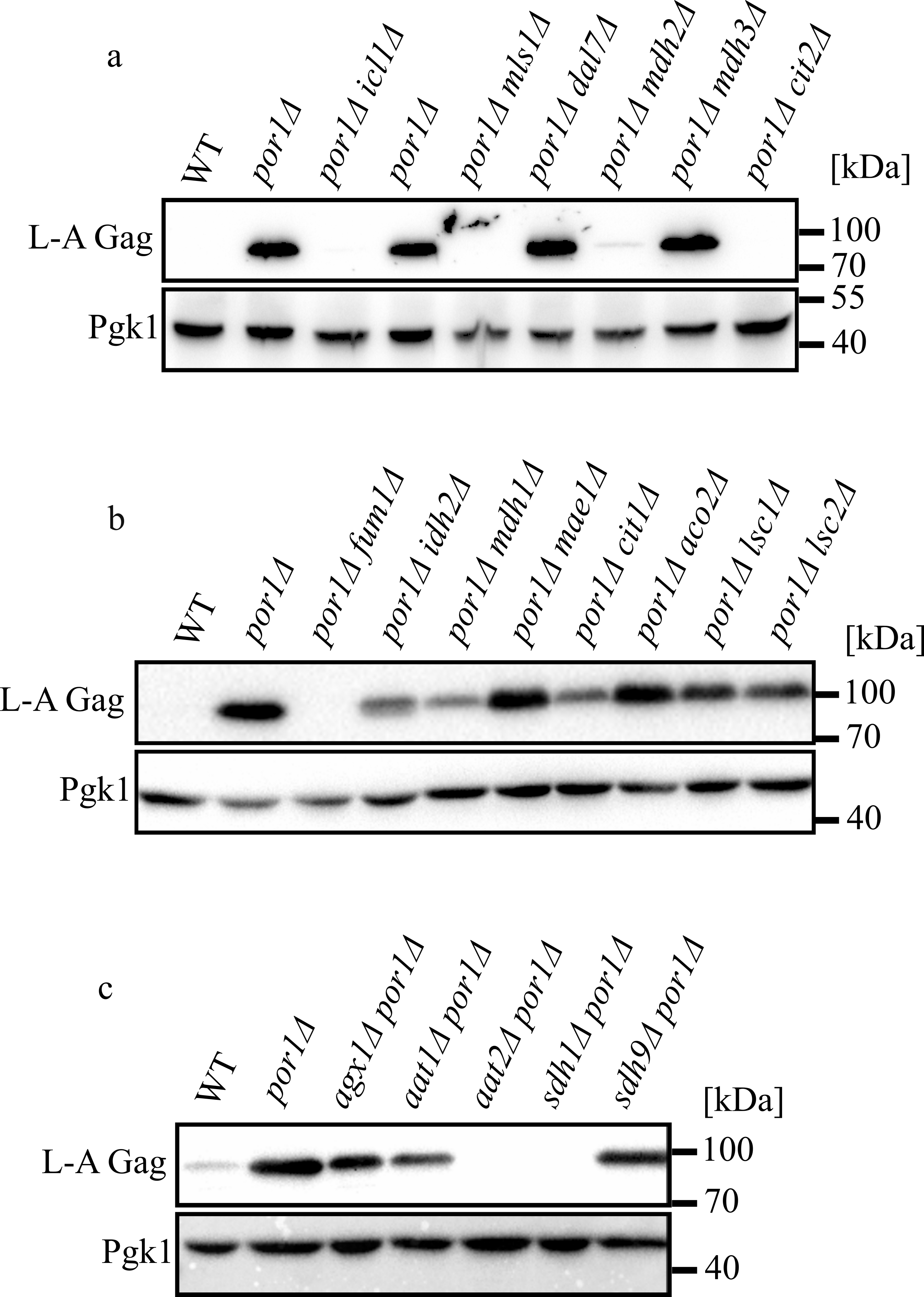

Supplement: iyag106_Supplementary_Data [file iyag106_supplementary_data.zip › Figure_S3_GENETICS-2026-309123.tif]

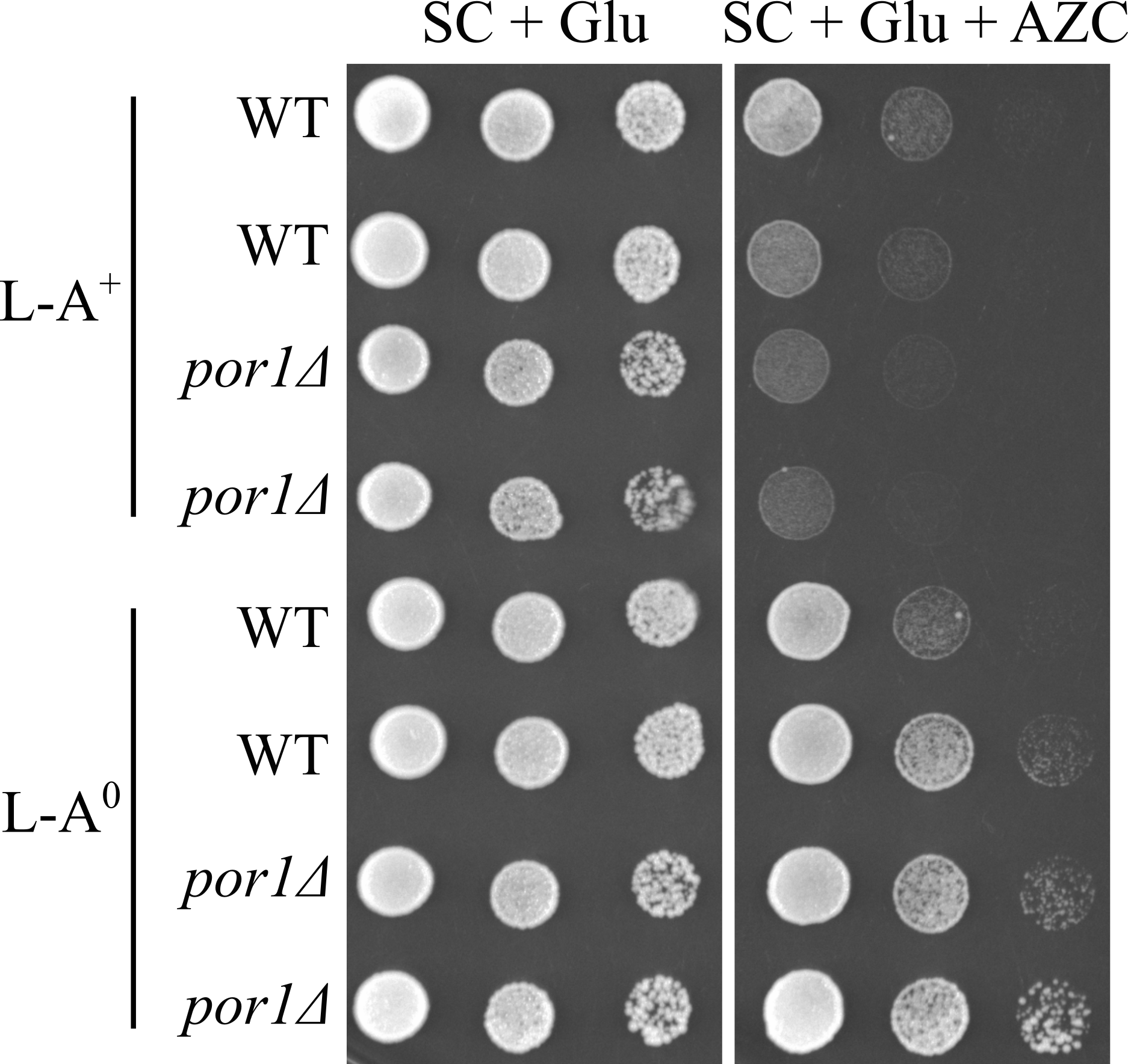

Supplement: iyag106_Supplementary_Data [file iyag106_supplementary_data.zip › Figure_S4_GENETICS-2026-309123.tif]
